# lcsm: An R package and tutorial on latent change score modelling

**DOI:** 10.12688/wellcomeopenres.17536.1

**Published:** 2022-05-11

**Authors:** Milan Wiedemann, Graham Thew, Urška Košir, Anke Ehlers

**Affiliations:** 1 Department of Experimental Psychology, University of Oxford, Oxford, UK; 2 Oxford Health NHS Foundation Trust, Oxford, UK; 3 Oxford University Hospitals NHS Foundation Trust, Oxford, UK

**Keywords:** latent change score modelling, structural equation modelling, longitudinal data analysis, lavaan, R

## Abstract

Latent change score models (LCSMs) are used across disciplines in behavioural sciences to study how constructs change over time. LCSMs can be used to estimate the trajectory of one construct (univariate) and allow the investigation of how changes between two constructs (bivariate) are associated with each other over time. This paper introduces the R package lcsm, a tool that aims to help users understand, analyse, and visualise different latent change score models. The lcsm package provides functions to generate model syntax for basic univariate and bivariate latent change score models with different model specifications. It is also possible to visualise different model specifications in simplified path diagrams. An interactive application illustrates the main functions of the package and demonstrates how the model syntax and path diagrams change based on different model specifications. This R package aims to increase the transparency of reporting analyses and to provide an additional resource to learn latent change score modelling.

## Introduction

Many psychological theories make predictions about changes over time and testing these predictions using appropriate statistical models is important to evaluate the evidence of these theories. Different statistical methods have been used to investigate changes in psychological constructs over time [for reviews see 1, 2]. Longitudinal structural equation modelling has been particularly popular to examine longitudinal processes because of its flexibility to build statistical models that match a particular psychological theory.

Latent change score models (LCSMs) are used across disciplines in the behavioural sciences to study how constructs change over time [e.g., 3– 6]. This framework can be extended to specifically examine how changes in one construct are associated with changes in another construct ^7^, and can be expanded to estimate more complex non-linear trajectories compared to other statistical techniques (e.g., autoregressive cross-lagged models). Such ‘change-to-change’ questions may be of interest across a range of research disciplines, given they can be used to examine whether changes on a predictor variable are related to changes on an outcome variable at a subsequent time point. In research on psychological therapies for example, a common question is whether changes in a given therapy process (e.g., negative appraisals) are related to subsequent changes on a therapy outcome measure (e.g., post-traumatic stress disorder (PTSD) symptoms). Latent change score modelling may be particularly suitable to study such questions. However, the most appropriate statistical model for evaluating change depends on the underlying theoretical model of change [see 2, 8, 9, for discussion].

This paper introduces the R package *lcsm*, a tool that aims to help users understand, analyse, and visualise different LCSMs. Below we start by providing a brief overview of LCSM methodology. For those seeking further introductory resources on structural equation modelling and the use of latent variables, we recommend Grimm *et al.* ^10^, Little ^11^, or Kline ^12^.

### Methodological overview

Latent change score modelling builds on concepts from classical test theory, which assumes that the observed score ( *X*) of an individual ( *i*) at a particular time ( *t*) can be expressed as the individual’s ‘true score’ ( *lx*) and the individual’s ‘unique score / residual’ ( *u*) at that time, see Equation 1. By dividing the observed scores in this way, models are able to test theories about unobserved (latent) constructs while reducing the effect of measurement residuals.


$$\;X_{ti}=lx_{ti}+u_{ti}\;(1)$$

The specification in LCSMs is such that the model can estimate a latent variable that captures the change in latent ‘true scores’ between two time points. By combining two longitudinal structural equation modelling methods, namely ‘latent growth curve models’ and ‘autoregressive cross-lag models’, LCSMs can provide a detailed examination of within-person changes in one (i.e., univariate) or more constructs (e.g., bivariate) over time ^10, 13^. A common modelling approach is to first understand the individual trajectories of each construct in a univariate LCSM, before combining both models to examine their relationships in a bivariate LCSM. The notation of the parameters in this paper mainly follow existing tutorials [e.g., 7, 10].

### Univariate LCSM

**Figure 1.**
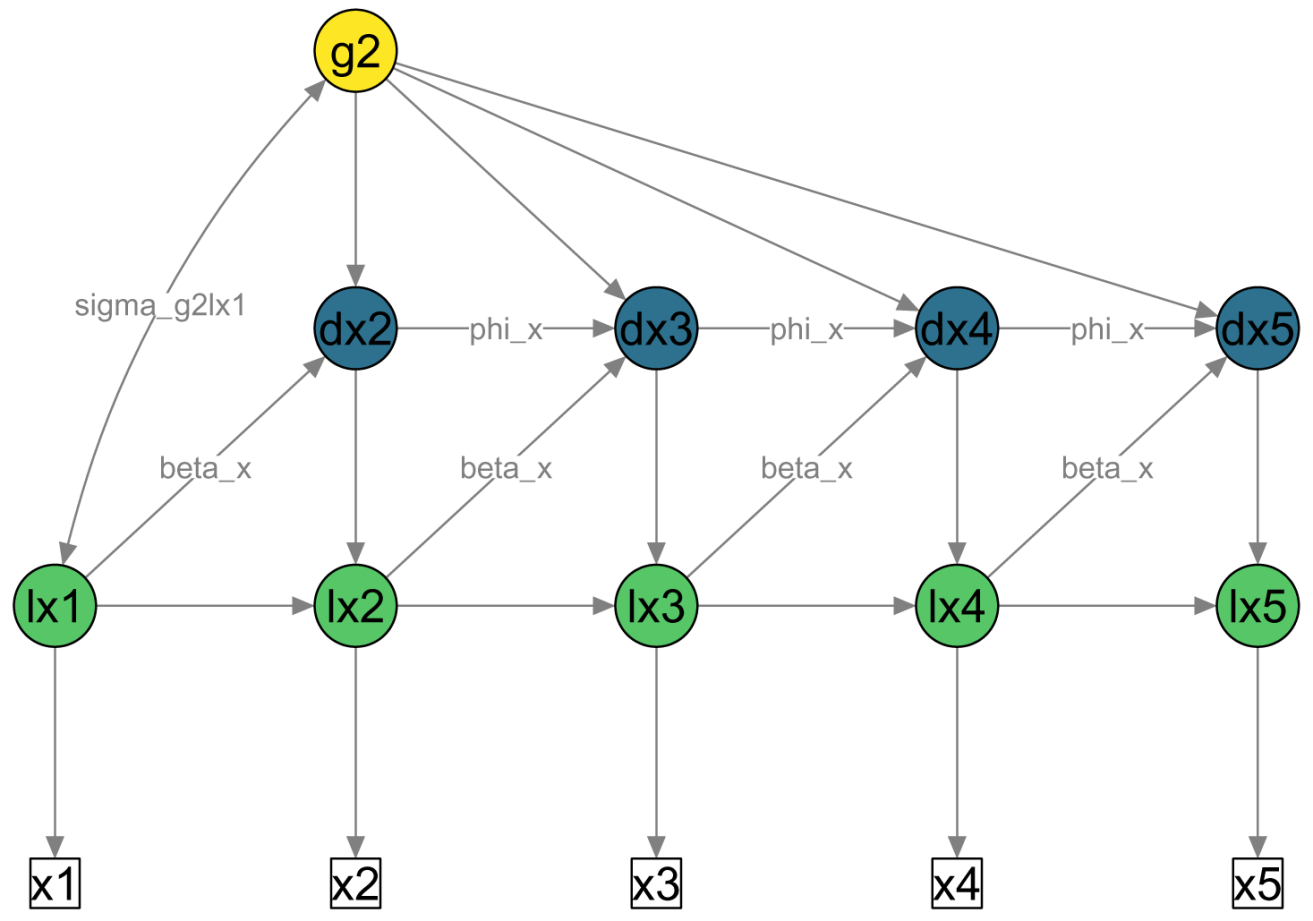
Simplified path diagram of univariate LCSM. White squares = Observed scores on variable ’x’ across timepoints 1 to 5; Green circles = Latent true scores (prefix ‘l’); Blue circles = Latent change scores (prefix ‘d’); Yellow circle = Constant latent change factor. Single-headed arrows = Regressions; Double-headed arrows = Covariance. beta_x = Proportional change factor; phi_x = Autoregression of change scores; sigma_g2lx1 = Covariance of change factor ( g2) with initial true score ( lx1). Unique scores ( *ux _t_*) and unique variances ( $\sigma_{ux}^{2}$ ) are not shown in this figure for simplicity.

A univariate LCSM aims to describe the changes of individuals ( *i*) in one construct ( *X*) over time ( *t*). The LCSM framework offers different options to describe this change: a constant change parameter ( *α _x_* × *s _xi_*), a proportional change parameter ( *β _x_* × *x*_[ *t*−1] _*i*__), and an autoregressive effect of the change scores ( *ϕ _x_* × ∆ *x*_[ *t*−1] _*i*__). The choice of which change components to include depends on the theoretical framework and model fit. Equation 2 shows how the change in one construct ( *X*) at a specific time point ( *t*) is specified when using these three parameters. The constant change parameter alone is similar to linear change because it has the same effect on all change scores (see the identical paths leading from g2 to each change score dx2 to dx5 in Figure 1). Proportional change describes whether the ‘change score’ at time ( *t*) is determined by the ‘true score’ of the same construct at the previous time point ( *t* − 1), see the paths labelled beta_x in Figure 1. Autoregressions of the change scores describe whether any given change score is determined by the previous change score (see the paths labelled phi_x in Figure 1). Note that in this example all parameters are constrained to be equal over time.


$$\text{Δ}x_{{\left[t\right]}_{i}}=\alpha_{x}\times s_{xi}+\beta_{x}\times x_{{\left[t-1\right]}_{i}}+\phi_{x}\times \text{Δ}x_{{\left[t-1\right]}_{i}}\;(2)$$

### Bivariate LCSM

The univariate LCSM can be extended to a bivariate LCSM to examine associations between two constructs over time. Depending on the research question there are different ‘coupling’ options to model the associations between two constructs [for a review see 10]. The following overview focuses on the extension that allows the examination of how changes in one construct are associated with changes in another construct ^7^. This particular bivariate LCSM can address research questions like: ‘Do *changes* in negative appraisals precede subsequent *changes* in PTSD symptoms during therapy?’.

**Figure 2.**
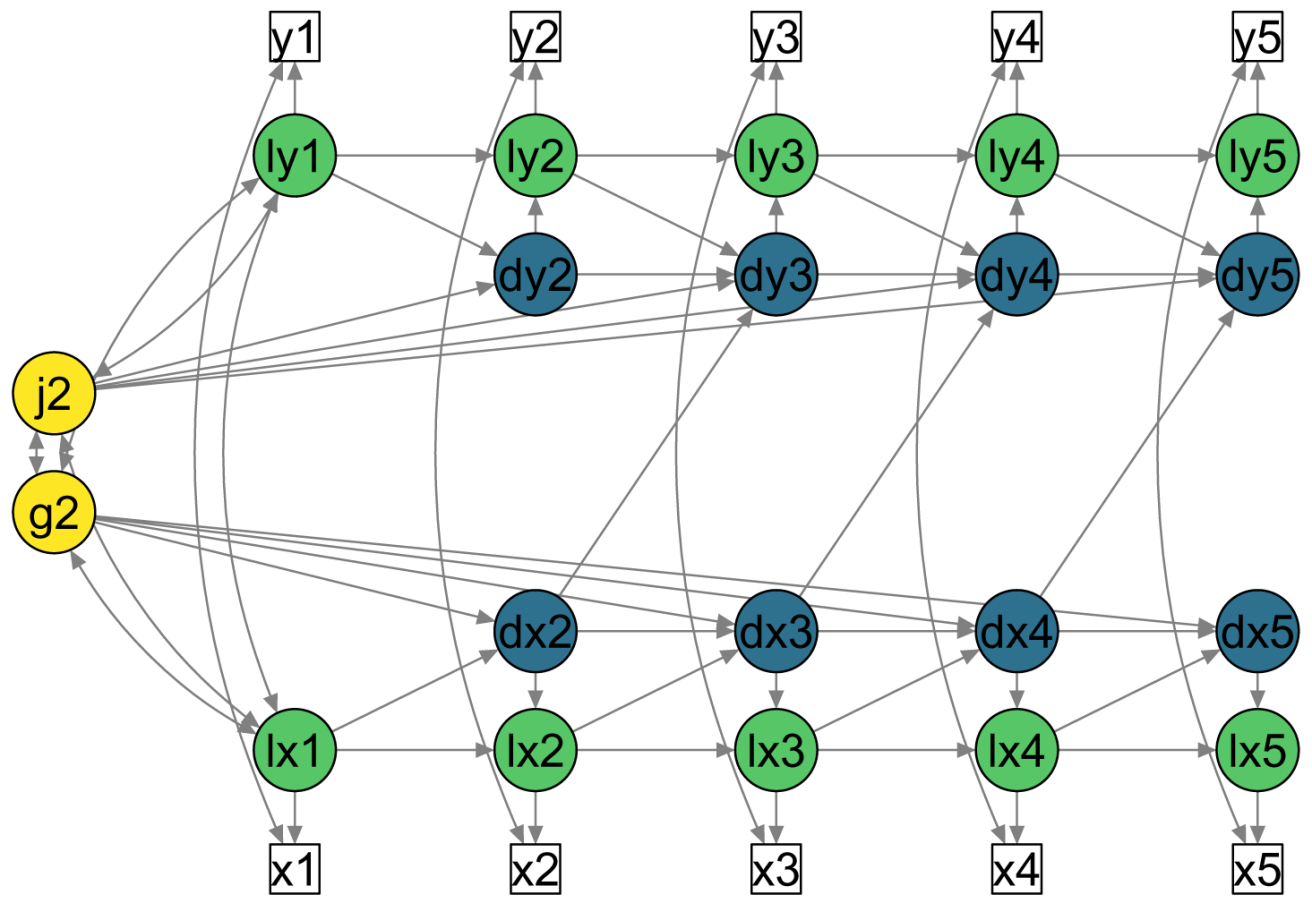
Simplified path diagram of bivariate LCSM with lagged change to change coupling parameters (e.g., dx2 to dy3). White squares = Observed variables; Green circles = Latent true scores (prefix ‘l’); Blue circles = Latent change scores (prefix ‘d’); Yellow circles = Constant latent change factors. Single-headed arrows = Regressions; Double-headed arrows = Covariance. Unique scores ( *ux _t_* and *uy _t_*) and unique variances ( $\sigma_{ux}^{2}$ and $\sigma_{uy}^{2}$ ) are not shown in this figure for simplicity.

A simplified path diagram of a bivariate LCSM with five repeated measurements and these parameters is shown in Figure 2. Equation 3 shows how changes of a bivariate LCSM with lagged coupling parameters are constructed. The first line of each equation represents the parameters that describe the within construct changes, while the second line of each equation represents parameters that describe the between construct coupling parameters. In this case, change in construct *X* is specified the same as in the univariate model, see Equation 3a. Change in construct *Y* is also specified using the ‘constant change’, ‘proportional change’, and ‘autoregressions of the change scores’ components but includes an additional element: the change in the other construct ( *X*) at the previous time point ( *ξ*_lag _*yx*__ × ∆ *x*_[ *t*−1] _*i*__), see Equation 3b. The parameter *ξ*_lag _*yx*__ × ∆ *x*_[ *t*−1] _*i*__ estimates whether changes in construct *Y* at one time point ( *t*) are determined by changes in construct *X* at the previous time point ( *t-1*).


$$\text{Δ}x_{{\left[t\right]}_{i}}=\alpha_{x}\times s_{xi}+\beta_{x}\times x_{{\left[t-1\right]}_{i}}+\phi_{x}\times \text{Δ}x_{{\left[t-1\right]}_{i}}\;(3a)$$

$$\begin{array}{l} \text{Δ}y_{{\left[t\right]}_{i}}=\alpha_{y}\times s_{yi}+\beta_{y}\times y_{{\left[t-1\right]}_{i}}+\phi_{y}\times \text{Δ}y_{{\left[t-1\right]}_{i}}+\\ \;\xi_{{\text{lag}}_{yx}}\times \text{Δ}x_{{\left[t-1\right]}_{i}} \end{array}\;(3b)$$

It is also important to consider whether to examine concurrent or lagged relationships between the two constructs. In some cases it may be desirable to examine concurrent relationships, for example when the underlying theory predicts that both constructs change simultaneously. For a more detailed discussion on this topic see Wang *et al.* ^14^ and Goldsmith *et al.* ^15^. Coefficients are usually constrained to be equal over all time points, but this can be changed by allowing variation between specific (or all) time points. For example, for an intervention with two distinct phases, the associations between two constructs might theoretically be thought to differ between phases, so coefficients could be constrained to be equal within each phase, but permitted to vary between phases. The *lcsm* package refers to this as a ‘piecewise’ approach.

### Why is a package needed?

Multiple script-based software packages support structural equation modelling and can be used for analysing LCSMs, for example *lavaan* ^16^, *OpenMx* ^17^, or *Mplus* ^18^. Other software packages like *JASP* ^19^ or *Ω*nyx ^20^ also offer a graphical user interface for building and analysing structural equation models. The R package *RAMpath* ^21^ offers a framework for analysing longitudinal structural equation models and it can also estimate basic univariate and bivariate LCSMs. Although there exist many tutorials on how to implement latent change score models in different software packages [e.g., 4, 10, 22– 24], most require the user to generate their own syntax from scratch, or manually adapt existing examples to match their data and model specifications. To our knowledge, there is currently no tool that allows the user to input different model specifications and automatically generate the corresponding LCSM syntax.

 Specifying LCSMs in current software packages can be complex and cumbersome, especially with larger numbers of repeated measures and when testing sequential models of increasing complexity. Syntax for complex models can be hard to program, is lengthy, and thus prone to errors. For example, *lavaan* syntax for a bivariate LCSM with 10 repeated measures can consist of between 200 to 300 lines of code. Klopack and Wickrama ^24^ also highlighted this drawback in a recent tutorial on latent change score modelling in *Mplus*, *‘Models can be cumbersome to program in available software packages.’* (p. 100). A tool that generates syntax for different model specifications may not only help to streamline the analytic steps involved in latent change score modelling, but also help researchers to reduce errors in code and facilitate a transparent and reproducible way of reporting analyses.

## Methods

### Implementation

The *lcsm* package combines the strengths of existing R packages for SEM by providing a framework that makes these packages work together efficiently. The current version of the package (Version 0.1.6) ^25^ provides a set of functions that help with visualising longitudinal data, generating *lavaan* syntax for different univariate and bivariate LCSMs, fitting univariate and bivariate LCSMs, and extracting parameter estimates as well as fit statistics.

Further functions allow plotting of simplified path diagrams and data simulation to explore the effects of different model parameters. An overview of the main functions and a short description is presented in Table 1.

**Table 1.** Main functions of the *lcsm* R package and their dependencies.

Function	Description	Main dependency ^‡^
**Specify lavaan syntax**		
specify_uni_lcsm()	Specify *lavaan* syntax for univariate LCSM	*base*
specify_bi_lcsm()	Specify *lavaan* syntax for bivariate LCSM	*base*
**Fit models**		
fit_uni_lcsm()	Fit univariate LCSM	*lavaan*
fit_bi_lcsm()	Fit bivariate LCSM	*lavaan*
**Extract results**		
extract_fit()	Extract fit statistics from *lavaan* objects	*broom*
extract_param()	Extract parameter estimates from *lavaan* objects	*broom*
**Simulate data**		
sim_uni_lcsm()	Simulate data from univariate LCSM parameters	*lavaan*
sim_bi_lcsm()	Simulate data from bivariate LCSM parameters	*lavaan*
**Visualisation functions**		
plot_trajectories()	Plot individual trajectories of cases	*ggplot2*
plot_lcsm()	Plot simplified LCSM path diagram	*semPlot*

*Note.* More details about each function can be found in the package documentation or using the help() function in R. ^‡ ^This column lists additional R packages that are required by the functions of the *lcsm* package.

### Operation

The *lcsm* package can be used with R version 3.5.0 or later on Linux, Mac and Windows. It can be installed from within R using install.packages("lcsm"). The interactive application *shinychange* supplements this package and illustrates how the *lavaan* syntax and path diagrams change depending on different model specifications. The next section gives an overview of the workflow of the *lcsm* package.

## Use cases

To demonstrate the implementation of these functions, we will consider the example of 500 participants undertaking a five session longitudinal intervention, where a hypothesised process variable (X), and a hypothesised outcome variable (Y), are measured at each session. Rather than simply looking at whether the process variable predicts outcomes at the end of the intervention, the LCSM approach allows the more detailed analysis of changes in each variable from one session to the next. In this example, our principal question is: Does the amount of *change* on the process variable X from one session to the next predict the amount of subsequent *change* in the outcome variable Y?

### Simulate data

Simulating data can be very helpful in order to learn new analytic approaches and refine study methodology as required ^26^. The functions sim_uni_lcsm() and sim_bi_lcsm() can be used to simulate data based on specific model parameter values specified by the user. Both of these functions return a dataframe in ’wide’ format ^1^ including a unique identifier ( id) for each individual. Note that LCSMs generally contain multiple parameters which are interdependent. It may not therefore be possible to specify the exact arrangement of parameters desired when simulating data.

In this example, we start by simulating a 500-participant dataset ( df_sim) for variables X and Y for the five session intervention, where both variables are expected to decrease over time and include some missing data. We are also specifying there should be a coupling relationship where change in X predicts subsequent change in Y using the coupling argument. The strength of this coupling relationship and other covariances describing associations between variables X and Y are specified in a list() using the coupling_param argument. All model parameters (e.g., model_x) are described in Table 2 and details about the parameter estimates (e.g., model_x_param) are presented in Table 3.


# Simulate data for worked example
df_sim <- sim_bi_lcsm(timepoints = 5, sample.nobs = 500,
                         # Specify percentage (pct) of missing data
                         na_x_pct = .15, na_y_pct = .1,
                         # Define parameters in model X
                         model_x = list(alpha_constant = TRUE, beta = TRUE, phi = TRUE),
                         # Specify parameter estimates in model X
                         model_x_param = list(gamma_lx1 = 29, sigma2_lx1 = .5,
                                                 sigma2_ux = .2, alpha_g2 = -.3,
                                                 sigma2_g2 = .6, sigma_g2lx1 = .2,
                                                 beta_x = -.1, phi_x = .1),
                         # Define parameters in model Y
                         model_y = list(alpha_constant = TRUE, beta = TRUE, phi = TRUE),
                         # Specify parameter estimates in model Y
                         model_y_param = list(gamma_ly1 = 15, sigma2_ly1 = .2,
                                                 sigma2_uy = .2, alpha_j2 = -.4,
                                                 sigma2_j2 = .1, sigma_j2ly1 = .02,
                                                 beta_y = -.2, phi_y = .1),
                         # Define coupling parameters
                         coupling = list(xi_lag_yx = TRUE),
                         # Specify coupling parameter estimates
                         coupling_param = list(sigma_su = .01, sigma_ly1lx1 = .2,
                                                  sigma_g2ly1 = .1, sigma_j2lx1 = .1,
                                                  sigma_j2g2 = .01, xi_lag_yx = .5),
                         # Set seed parameter
                         seed = 1234)


**Table 2.** Available specifications for univariate LCSMs and bivariate coupling options.

Option	Description
Univariate model options	
alpha_constant	Constant change factor
alpha_piecewise	Piecewise constant change factor
alpha_piecewise_num	Change point of piecewise constant change factor
alpha_linear	Linear change factor
beta	Proportional change factor: change score x (t) determined by true score x (t-1)
phi	Autoregression of change scores: change score x (t) determined by change score x (t-1)
**Coupling options**	
coupling_piecewise	Piecewise coupling parameters
coupling_piecewise_num	Change point of piecewise coupling parameters
delta_con_xy	Change score x (t) determined by true score y (t)
delta_con_yx	Change score y (t) determined by true score x (t)
delta_lag_xy	Change score x (t) determined by true score y (t-1)
delta_lag_yx	Change score y (t) determined by true score x (t-1)
xi_con_xy	Change score x (t) determined by change score y (t)
xi_con_yx	Change score y (t) determined by change score x (t)
xi_lag_xy	Change score x (t) determined by change score y (t-1)
xi_lag_yx	Change score y (t) determined by change score x (t-1)

*Note.* Covar =Covariance. More details about each model option as well as further customisations can be found in the package documentation using help(specify_uni_lcsm) or help(specify_bi_lcsm). Bivariate model options allow for concurrent ( con) and lagged ( lag) coupling between two constructs.

**Table 3.** Complete list of parameters available in the *lcsm* package.

Parameter	Symbol	Description
**Construct X **		
gamma_lx1	*γ _lx_*_1_	Mean of latent true scores x (Intercept)
sigma2_lx1	$\sigma_{lx1}^{2}$	Variance of latent true scores x
sigma2_ux	$\sigma_{ux}^{2}$	Variance of observed scores x
alpha_g2	*α*_*g* ^2^_	Mean of change factor (g2)
alpha_g3	*α*_*g* ^3^_	Mean of change factor (g3)
sigma2_g2	$\sigma_{g2}^{2}$	Variance of change factor (g2)
sigma2_g3	$\sigma_{g3}^{2}$	Variance of change factor (g3)
sigma_g2lx1	*σ*_*g*2 *lx*1_	Covar: Change factor (g2) with initial true score x (lx1)
sigma_g3lx1	*σ*_*g*3 *lx*1_	Covar: Change factor (g3) with initial true score x (lx1)
sigma_g2g3	*σ*_*g*2 *g*3_	Covar: Change factors within construct x
beta_x	*β _x_*	Proportional change factor of construct x
phi_x	*ϕ _x_*	Autoregression of change scores x
**Construct Y**		
gamma_ly1	*γ*_*ly*1_	Mean of latent true scores y (Intercept)
sigma2_ly1	$\sigma_{ly1}^{2}$	Variance of latent true scores y
sigma2_uy	$\sigma_{uy}^{2}$	Variance of observed scores y
alpha_j2	*α*_*j*2_	Mean of change factor (j2)
alpha_j3	*α*_*j*3_	Mean of change factor (j3)
sigma2_j2	$\sigma_{j2}^{2}$	Variance of change factor (j2)
sigma2_j3	$\sigma_{j3}^{2}$	Variance of change factor (j3)
sigma_j2ly1	*σ*_*j*2 *ly*1_	Covar: Change factor (j2) with initial true score y (ly1)
sigma_j3ly1	*σ*_*j*3 *ly*1_	Covar: Change factor (j3) with initial true score y (ly1)
sigma_j2j3	*σ*_*j*2 *j*3_	Covar: Change factors within construct y
beta_y	*β _y_*	Proportional change factor of construct y
phi_y	*ϕ _y_*	Autoregression of change scores y
**Coupling X & Y**		
sigma_su	*σ _su_*	Covar: Residuals x with y
sigma_ly1lx1	*σ*_*ly*1 *lx*1_	Covar: Intercepts x with y
sigma_g2ly1	*σ*_*g*2 *ly*1_	Covar: Change factor x (g2) with initial true score y (ly1)
sigma_g3ly1	*σ*_*g*3 *ly*1_	Covar: Change factor x (g3) with initial true score y (ly1)
sigma_j2lx1	*σ*_*j*2 *lx*1_	Covar: Change factor y (j2) with initial true score x (lx1)
sigma_j3lx1	*σ*_*j*3 *lx*1_	Covar: Change factor y (j3) with initial true score x (lx1)
sigma_j2g2	*σ*_*j*2 *g*2_	Covar: Change factors y (j2) with x (g2)
sigma_j2g3	*σ*_*j*2 *g*3_	Covar: Change factors y (j2) with x (g3)
sigma_j3g2	*σ*_*j*3 *g*2_	Covar: Change factors y (j3) with x (g2)
delta_con_xy	*δ*_con *xy*_	Change score x (t) determined by true score y (t)
delta_con_yx	*δ*_con *yx*_	Change score y (t) determined by true score x (t)
delta_lag_xy	*δ*_lag *xy*_	Change score x (t) determined by true score y (t-1)
delta_lag_yx	*δ*_lag *yx*_	Change score y (t) determined by true score x (t-1)
xi_con_xy	*ξ*_con *xy*_	Change score x (t) determined by change score y (t)
xi_con_yx	*ξ*_con *yx*_	Change score y (t) determined by change score x (t)
xi_lag_xy	*ξ*_lag *xy*_	Change score x (t) determined by change score y (t-1)
xi_lag_yx	*ξ*_lag *yx*_	Change score y (t) determined by change score x (t-1)

*Note.* Covar = Covariance. More details for each parameter can be found in the package documentation using help(sim_uni_lcsm) or help(sim_bi_lcsm).

### Visualise longitudinal data

Simulated or real datasets can then be visualised to help with exploring and understanding how the model parameters are associated with different trajectories of change over time. Visualising individual trajectories of repeated measurements may also be helpful for understanding data and informing modelling decisions. A more detailed overview of the rationale for visualising longitudinal data is described in Ghisletta and McArdle ^23^ and Grimm *et al.* ^10^.

**Figure 3.**
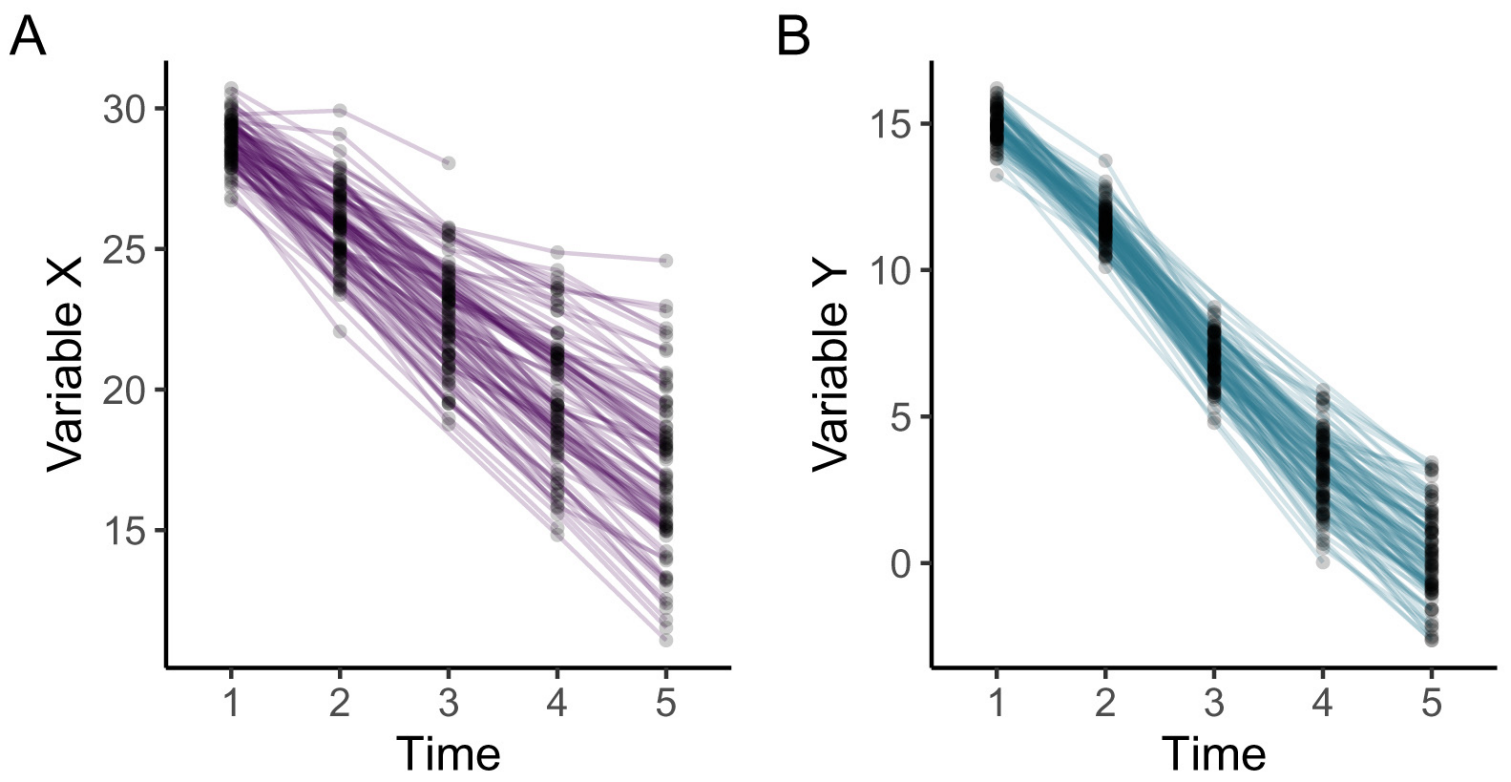
Longitudinal plots of five repeated measurements of example variables X and Y.

The function plot_trajectories() offers an easy way to visualise longitudinal data. Figure 3 was created using this function to visualise a random 20% sample of individual participants’ trajectories on variables X and Y in our example dataset ( df_sim). This function is built using the R package *ggplot2* ^27^ and additional *ggplot2* functions can be added to extend the plot.

### Fit univariate and bivariate models

Having visualised our example data, we now aim to implement univariate models for variables X and Y separately, before combining these together in a bivariate model. The functions fit_uni_lcsm() and fit_bi_lcsm() can be used to do this, using the *lavaan* package ^16^. By default, full information maximum likelihood (FIML) is used to estimate each model. This method makes the assumption that data is missing completely at random (MCAR) or missing at random (MAR), and permits the inclusion of individuals with incomplete data ^28^. Other model estimators and methods to treat missing data that are part of the *lavaan* package can be specified using the two arguments estimator and missing (for more details see help(lavOptions)).

The example below shows a univariate model for the process variable X. The user specifies the dataset in wide format using the 'data' argument. A list of variables representing the repeated measures of X are specified in the 'var' argument. The LCSM parameters are specified as a list() using the 'model' argument. For example, the code below includes a constant change factor ( alpha_constant), a proportional change factor ( beta), and the autoregression of change scores ( phi). Figure 1 shows a simplified path diagram of this model.


# Fit univariate latent change score model and
# save the returned object as 'model1'
model1 <- fit_uni_lcsm(data = df_sim,
                          var = c("x1", "x2", "x3", "x4", "x5"),
                          model = list(alpha_constant = TRUE,
                                         beta = TRUE,
                                         phi = TRUE))


Once we have specified univariate models for both variable X and variable Y, and have evaluated the extent to which these models fit the data using fit statistics (described below), we can combine the best-fitting univariate models into a bivariate LCSM using the function fit_bi_lcsm(). The below example shows this model. The two sets of repeated measures are specified using the arguments 'var_x' and 'var_y', followed by the specifications of the best-fitting univariate models ( 'model_x' and 'model_y'). Note that in this example the specifications of these two models use identical parameters ( alpha_constant, beta, and phi). However, nonidentical specifications for 'model_x' and 'model_y' can be made if this is indicated by the model fit of the individual univariate models.

For bivariate LCSMs, ‘coupling’ parameters, which model the interactions between variables X and Y, can be specified using the 'coupling' argument. The argument shown below, 'coupling = list(xi_lag_yx = TRUE)', addresses the main question of our worked example, by adding a parameter that estimates whether changes in variable *Y* at time point ( *t*) are determined by changes in variable *X* at the previous time point ( *t* − 1). Figure 2 shows a simplified path diagram of this model.


# Fit bivariate latent change score model and
# save the returned object as 'model2'
model2 <- fit_bi_lcsm(data = df_sim,
                         var_x = c("x1", "x2", "x3", "x4", "x5"),
                         var_y = c("y1", "y2", "y3", "y4", "y5"),
                         model_x = list(alpha_constant = TRUE,
                                          beta = TRUE, phi = TRUE),
                         model_y = list(alpha_constant = TRUE,
                                          beta = TRUE, phi = TRUE),
                         coupling = list(xi_lag_yx = TRUE))


If the time lag element ( lag) is not desired, a concurrent ( con) version of each coupling parameter is available (e.g., 'coupling = list(xi_con_yx = TRUE)'). Table 2 shows the full list of available univariate and bivariate model specifications that are implemented in the current version of the *lcsm* package. These specifications can be included in the LCSM by adding them to the list of parameters and setting them to TRUE.

**Figure 4.**
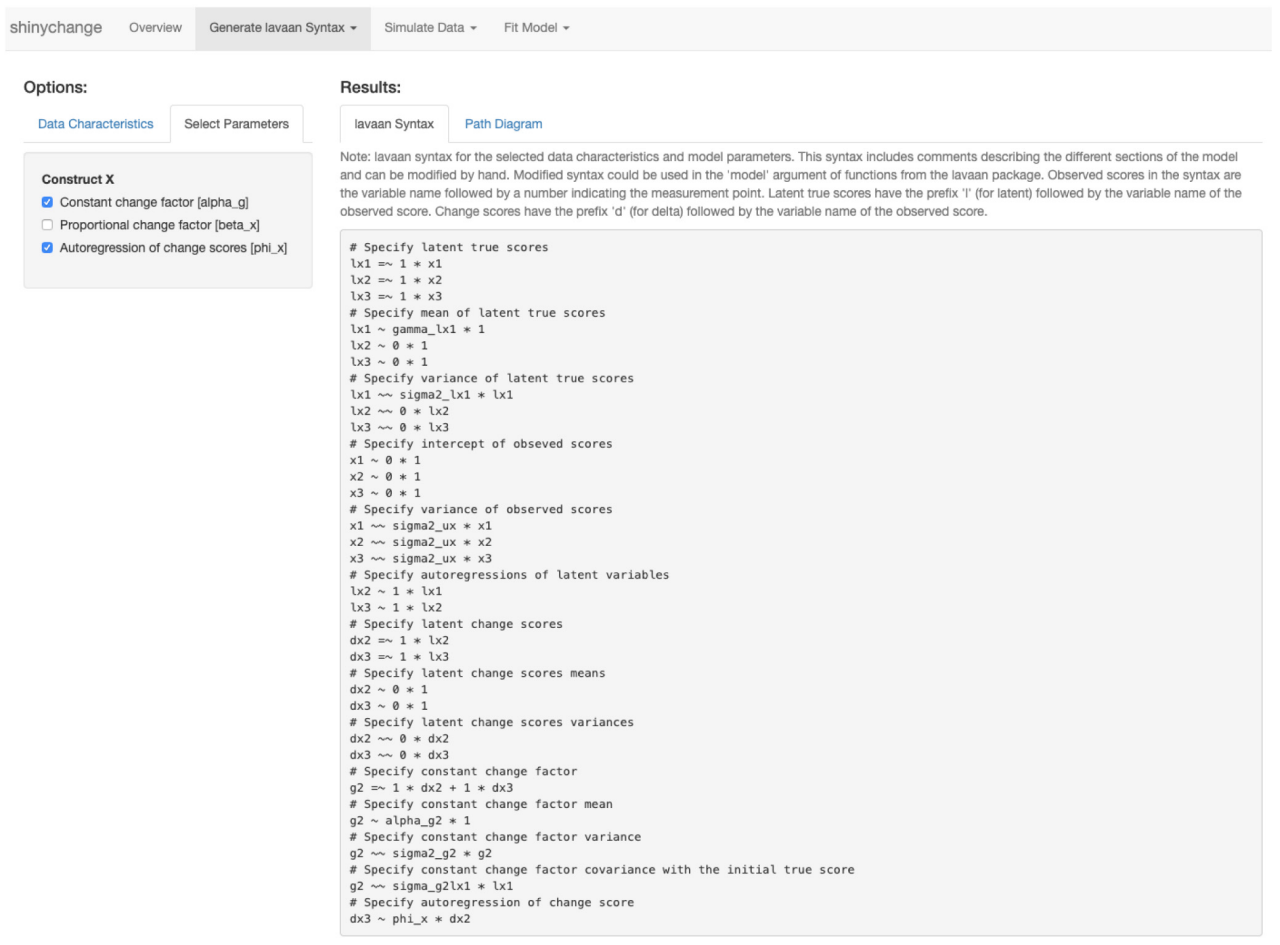
An example of generating *lavaan* syntax of a univariate LCSM using *shinychange*.

For users who wish only to generate the *lavaan* syntax of a model, the functions specify_uni_lcsm() and specify_bi_lcsm() are available. This may be useful to those who wish to make further manual adaptations to the model syntax (e.g., freeing parameters that are fixed over time by default). The *lcsm* package adds comments to the *lavaan* syntax to provide more information about each section and to facilitate such adaptations. The interactive application *shinychange* allows users to explore how the number of repeated measures and different parameters affect the *lavaan* syntax (see Figure 4).

### Extract fit statistics and parameter estimates

To evaluate how well a univariate or bivariate model fits the underlying data, one option is to compare the fit statistics of differently specified models [for further discussion of approaches to evaluating model fit see 10]. This is usually performed in a ‘nested’ way, comparing similar models of increasing complexity. Imagine we wish to compare two versions of a univariate model for the outcome variable *Y*. We want to evaluate whether the addition of the ‘autoregression of change scores’ parameter ( phi) in the model provides a better description of the data. To do this, we would estimate two nested models; model_y_01, which does not include phi, and model_y_02, which includes phi. The models are otherwise identical. The function extract_fit(model_y_01, model_y_02) can be used to extract commonly used fit statistics for each model. These can be inspected visually or the two models can be compared using the anova() function from *lavaan* to perform a likelihood ratio test. Below is an example output, using the univariate model model1 we created earlier.


# Extract fit statistics from 'model1' created earlier
# More fit statistics can be displayed changing the details argument to TRUE
extract_fit(model1, details = FALSE)

# # # A tibble: 1 × 8
# #   model chisq  npar   aic   bic   cfi rmsea   srmr
# #   <chr> <dbl> <dbl> <dbl> <dbl> <dbl> <dbl>  <dbl>
# # 1     1  11.0     8 5471. 5504.     1     0 0.0814


Parameter estimates of any LCSM fitted with the *lcsm* package can be extracted using the extract_param() function, which builds on the R package *broom* ^29^. This function returns a dataframe listing each parameter included within the model, together with the corresponding estimate, standard error, and p value. These data can then be interpreted in relation to the study hypotheses. Names and descriptions of all parameters are presented in Table 3.


# Extract parameter estimates from 'model1' created earlier
# To simplify the output we change the formatting of p values
# and only select the first 5 variables
extract_param(model1, printp = TRUE) %>%
    select(1:5)

# # # A tibble: 8 × 5
# #   label       estimate std.error statistic p.value
# #   <chr>          <dbl>     <dbl>     <dbl>  <chr>
# # 1 gamma_lx1     28.9     0.0396    730.    < .001
# # 2 sigma2_lx1     0.528   0.0461     11.4   < .001
# # 3 sigma2_ux      0.194   0.00783    24.8   < .001
# # 4 alpha_g2       0.109   0.192       0.567   .571
# # 5 sigma2_g2      0.657   0.0443     14.8   < .001
# # 6 sigma_g2lx1    0.236   0.0332      7.11  < .001
# # 7 beta_x        -0.111   0.00603   -18.5   < .001
# # 8 phi_x          0.142   0.0184      7.68  < .001


### Plot simplified path diagrams

Visualising models using a simplified path diagram can be a helpful method to understand and check the models being specified, by giving a clear visual representation of the parameters chosen. The plot_lcsm() function of this package can be used to generate such diagrams, and is built on the *semPlot* package ^30^. Figure 1 and Figure 2 were both created using this function.

## Discussion

Analysing the longitudinal relationships between changes in two constructs may help to better understand how they unfold over time. LCSMs are a specific form of longitudinal structural equation models that allow researchers to examine these relationships. In psychotherapy research this can be used to examine how changes in specific therapy processes (e.g., negative appraisals) are associated with subsequent changes on a treatment outcome measure (e.g., PTSD symptoms).

This paper addresses a small subset of the specifications available using a latent change score modelling approach. Further adaptations may be required to address specific research questions. Grimm *et al.* ^10^ provide a more detailed overview of the methodological background to these models and discuss some possible adaptations and extensions. It is also important to mention that the interpretation of results from LCSMs needs to be considered carefully, especially when multiple parameters (e.g., constant change and proportional change) are used to examine change over time [see 31, 32]. It is hoped that the *lcsm* package and this tutorial will provide a helpful resource for understanding and implementing LCSMs, and aid in the clear and transparent reporting of these analyses.

## Data availability

All code, materials, and data can be found at https://github.com/milanwiedemann/lcsm. 

## Software availability

Source code available from: https://github.com/milanwiedemann/lcsm.

Archived source code at time of publication: https://zenodo.org/record/6451333#.YlfDZy8Rr0o ^25^.

License: MIT

Instructions for installing the package, further technical details, and examples can be found at https://milanwiedemann.github.io/lcsm.
