# Supplementary material for: lcsm: An R package and tutorial on latent change score modelling
Source: Wellcome Open Res. 2022 May 11;7:149. [Version 1] doi: 10.12688/wellcomeopenres.17536.1 (PMC9547120; doi:10.12688/wellcomeopenres.17536.1)
Supplement: Peer review history

# Peer review: Reviewer response for version 1

**DOI:** 10.21956/wellcomeopenres.19390.r52414

**Published:** 2022-10-07

**Authors:** Emil N. Coman

**Affiliations:** 1 School of Medicine, Health Disparities Institute, University of Connecticut, Hartford, CT, USA

This is an exciting development, to have a dedicated latent change/difference score package in R.

 Sorry for delaying the feedback; my suggestions refer mainly to how LCS is introduced and explained (briefly, as needed here, of course).

1. The LDS tool was invented by Jack McArdle long ago in 1991 (earliest I know of). ^1^ in a less known (and accessible) chapter, as a response to Wohlwill’s chapter in the same book, where such ideas were raised. I posted all McArdle chapters (and Wohlwill’s, not of much use) that I have here to cross-check my statements: https://www.dropbox.com/s/x6ljf29t1oa9any/McArdle_chapters.zip?dl=0
  Its use in change analyses is coupled with its use in difference analyses, see #3.
2. A simple way to explain and make the LDS/LCS insight is shown in this piece ^2^ (which you don’t need to cite, of course, unless you find info needed to include, and need to give credit for that): it is a rare instance of forcing a mathematical equality between statistical concepts in a model setup: it literally forces the second measure (post, T2; or person 2 in a dyad) to be the sum of the first (pre, T1; or person 1 in a dyad) and the change (LCS/LDS in dyadic setup) itself: Y2 = Y1 + LCS/LDS. This is achieved by means of a regression (better even path analytic or SEM) setup, in a model where most coefficients are fixed, to 1’s or 0’s.
3. LDS terminology (and tool) is used to some extent in ‘therapeutic alliance’ type works, e.g. ^3^; this should be mentioned too.
4. The fixing of some of the coefficients needed in the very definition of LCS/LDS latents is not easy to pull off in applied software implementations, e.g. in Stata (gsem module) the forcing of the residual variance of the second variable is not allowed (in the Stata sem module, this works). How R handles this setting therefore for categorical outcomes in particular might be a good detail to mention.

Are the conclusions about the tool and its performance adequately supported by the findings presented in the article?

Yes

Is the rationale for developing the new software tool clearly explained?

Yes

Is the description of the software tool technically sound?

Yes

Are sufficient details of the code, methods and analysis (if applicable) provided to allow replication of the software development and its use by others?

Yes

Is sufficient information provided to allow interpretation of the expected output datasets and any results generated using the tool?

Yes

Reviewer Expertise:

Path analysis, causality

I confirm that I have read this submission and believe that I have an appropriate level of expertise to confirm that it is of an acceptable scientific standard.

## References

[1] : Structural models of developmental theory in psychology, in Annals of Theoretical Psychology, P.V. Geert and L. Mos, Editors. *Plenum Publishers: New York* .1991;139-160.

[2] : The paired t-test as a simple latent change score model. *Front Psychol* .2013;4: 10.3389/fpsyg.2013.00738 738 10.3389/fpsyg.2013.00738 24124419PMC3794455

[3] : The relationship of perfectionism, depression, and therapeutic alliance during treatment for depression: latent difference score analysis. *J Consult Clin Psychol* .2006;74(5) : 10.1037/0022-006X.74.5.930 930-42 10.1037/0022-006X.74.5.930 17032097

# Peer review: Reviewer response for version 1

**DOI:** 10.21956/wellcomeopenres.19390.r52415

**Published:** 2022-09-29

**Authors:** Kevin J. Grimm

**Affiliations:** 1 Department of Psychology, Arizona State University, Tempe, AZ, USA

The authors developed and discuss an R package for specifying and visualizing latent change score models. The package appears to work as expected to generate lavaan code for the model. Some things for the authors to consider:

1. "By dividing" should be "By decomposing"
2.  I wouldn't say how change in one construct (X) as a specific time point (t) because X is the observed variable and LCSMs are evaluating changes at the latent level (i.e., lx). This happens throughout the manuscript.
3. Toward the end of page 2, I wouldn't begin discussing Figure 1 until the mathematical specification is complete.
4. There are several places where the authors are repeating (t) for time and (i) for individual.
5.  I wouldn't have a question as a header (i.e., Why is a package needed?). Be declarative or simply use "lcsm package" and then describe its need as you have done.

Are the conclusions about the tool and its performance adequately supported by the findings presented in the article?

Yes

Is the rationale for developing the new software tool clearly explained?

Yes

Is the description of the software tool technically sound?

Yes

Are sufficient details of the code, methods and analysis (if applicable) provided to allow replication of the software development and its use by others?

Yes

Is sufficient information provided to allow interpretation of the expected output datasets and any results generated using the tool?

Yes

Reviewer Expertise:

Longitudinal data analysis, machine learning, latent change score modeling

I confirm that I have read this submission and believe that I have an appropriate level of expertise to confirm that it is of an acceptable scientific standard.
